# Supplementary material for: Induction of a Compensatory Photosynthetic Response Mechanism in Tomato Leaves upon Short Time Feeding by the Chewing Insect Spodoptera exigua
Source: Insects. 2021 Jun 18;12(6):562. doi: 10.3390/insects12060562 (PMC8234478; doi:10.3390/insects12060562)
Supplement: Supplementary file 1 [file insects-12-00562-s001.zip › insects-1212232-supplementary.pdf]

Article

Induction of a Compensatory Photosynthetic Response Mechanism in Tomato Leaves Upon Short Time Feeding by the Chewing Insect *Spodoptera exigua*

Julietta Moustaka [1], Nicolai Vitt Meyling [1] and Thure Pavlo Hauser [1*]

Table S1. P-values of the paired Student's t test performed between the whole leaflet's AOIs at all different time points for the Quantum Yields: Φ_{PSII}, Φ_{NPQ}, Φ_{NO}.

Quantum Yields	Φ_{PSII}				Φ_{NPQ}				Φ_{NO}			
	Before	15 m	90 m	180 m	Before	15 m	90 m	180 m	Before	15 m	90 m	180 m
**Before**												
**15 m**	**5.80783E-05**				**2.45E-08**				0.283			
**90 m**	0.054	0.740			**1.52E-05**	0.096			**0.020**	**0.017**		
**180 m**	0.294	**0.004**	**2.17E-06**		0.858	**0.014**	**2.86E-11**		0.191	0.571	**1.5E-06**	

Table S2. P-values of the paired Student's t test performed between all different zones at all different time points for the Effective quantum yield of PSII photochemistry, Φ_{PSII}.

Φ_{PSII}	Before	15 m rest	15 m surrounding	15 m feeding spot	90 m rest	90 m surrounding	90 m feeding spot	180 m rest	180 m surrounding	180 m feeding spot
**15min rest**	**1.94E-07**									
**15min surrounding**	**0.004**	**0.029**								
**15min feeding**	**0.018**	**0.008**	**0.011**							
**90min rest**	**0.000**	0.656	0.452	**0.004**						
**90min surrounding**	**0.014**	**0.048**	0.889	**0.001**	0.539					
**90min feeding**	**0.050**	0.076	**0.011**	0.672	**0.025**	**0.022**				
**180min rest**	0.369	**0.012**	0.240	**0.005**	**3.56E-08**	0.092	**0.041**			
**180min surrounding**	0.237	0.690	0.213	0.353	**0.035**	**0.002**	**0.026**	0.367		
**180min feeding**	**0.049**	0.064	**0.003**	**0.003**	**0.027**	**0.015**	0.312	**0.047**	**0.027**	

Table S3. P-values of the paired Student's t test performed between all different zones at all different time points for the Quantum yield of regulated non photochemical energy loss in PSII, Φ_{NPQ}.

Φ_{NPQ}	**Before**	**15 m rest**	**15 m surrounding**	**15 m feeding spot**	**90 m rest**	**90 m surrounding**	**90 m feeding spot**	**180 m rest**	**180 m surrounding**	**180 m feeding spot**
**15m rest**	**2.56E-08**									
**15m surrounding**	**0.001**	**0.006**								
**15m feeding**	**0.002**	**0.004**	0.637							
**90 m rest**	**0.007**	0.461	0.540	0.983						
**90 m surrounding**	**0.002**	**0.010**	0.881	0.849	0.633					
**90 m feeding**	**0.010**	**0.011**	0.982	0.274	0.321	0.326				
**180 m rest**	0.252	**0.012**	0.090	0.146	**5.52E-10**	**0.024**	0.065			
**180 m surrounding**	**0.047**	0.388	0.135	0.138	**0.011**	**0.001**	0.177	0.525		
**180m feeding**	0.052	0.071	0.787	0.266	0.168	0.283	0.285	0.348	0.525	

Table S4. P-values of the paired Student's t test performed between all different zones at all different time points for the Quantum yield of non-regulated energy loss in PSII, Φ_{NO}.

Φ_{NO}	**Before**	**15 m rest**	**15 m surrounding**	**15 m feeding spot**	**90 m rest**	**90 m surrounding**	**90 m feeding spot**	**180 m rest**	**180 m surrounding**	**180 m feeding spot**
**15min rest**	0.673352									
**15min surrounding**	**1.37E-05**	**4.23E-04**								
**15min feeding**	**0.005**	**0.005**	**0.009**							
**90min rest**	0.537	0.158	0.906	**0.012**						
**90min surrounding**	**2.38E-04**	**0.002**	0.791	**0.014**	0.895					
**90min feeding**	**0.023**	**0.033**	**0.042**	0.489	**0.030**	**0.037**				
**180min rest**	0.187	**0.048**	**0.003**	**0.009**	**2.55E-06**	**0.007**	**0.028**			
**180min surrounding**	**0.002**	**0.043**	**0.021**	**0.009**	**0.043**	**0.014**	**0.031**	0.117		
**180min feeding**	**0.037**	0.055	0.073	0.270	0.051	0.067	0.095	**0.045**	0.052	

Table S5. P-values of the paired Student's t test performed between all different zones at all different time points for the Non Photochemical Quenching, NPQ.

NPQ	Before	15 m rest	15 m surrounding	15 m feeding spot	90 m rest	90 m surrounding	90 m feeding spot	180 m rest	180 m surrounding	180 m feeding spot
15min rest	6.71E-07									
15min surrounding	9.70E-05	0.002								
15min feeding	3.63E-04	0.001	0.047							
90min rest	0.024	0.327	0.594	0.094						
90min surrounding	0.000	0.003	0.865	0.088	0.735					
90min feeding	0.004	0.010	0.208	0.364	0.051	0.100				
180min rest	0.293	0.013	0.039	0.017	1.40E-10	0.011	0.011			
180min surrounding	0.007	0.183	0.094	0.014	0.007	0.001	0.031	0.883		
180min feeding	0.022	0.049	0.453	0.297	0.329	0.477	0.205	0.060	0.140	

Table S6. P-values of the paired Student's t test performed between all different zones at all different time points for the Electron Transport Rate, ETR.

ETR	Before	15 m rest	15 m surrounding	15 m feeding spot	90 m rest	90 m surrounding	90 m feeding spot	180 m rest	180 m surrounding	180 m feeding spot
15min rest	3.42E-09									
15min surrounding	0.004	0.029								
15min feeding	0.018	0.008	0.015							
90min rest	3.70E-04	0.658	0.452	0.005						
90min surrounding	0.013	0.048	0.885	0.001	0.549					
90min feeding	0.051	0.075	0.011	0.668	0.025	0.021				
180min rest	0.372	0.012	0.239	0.005	3.77E-08	0.095	0.042			
180min surrounding	0.238	0.694	0.213	0.003	0.036	0.002	0.026	0.373		
180min feeding	0.050	0.063	0.003	0.351	0.027	0.015	0.312	0.048	0.028	

Table S7. P-values of the paired Student's t test performed between all different zones at all different time points for the Efficiency of open PSII reaction centers Fv'/Fm'.

F_v'/F_m'	Before	15 m rest	15 m surrounding	15 m feeding spot	90 m rest	90 m surrounding	90 m feeding spot	180 m rest	180 m surrounding	180 m feeding spot
15min rest	**5.45E-05**									
15min surrounding	0.456	0.350								
15min feeding	0.064	0.075	**0.036**							
90min rest	**0.012**	**0.027**	0.281	0.155						
90min surrounding	0.112	0.099	0.329	**0.004**	0.181					
90min feeding	0.068	0.088	0.146	0.969	0.185	**0.031**				
180min rest	0.101	0.542	0.118	0.129	**3.62E-05**	0.067	0.175			
180min surrounding	0.790	0.628	0.808	**0.013**	0.536	**0.010**	0.054	0.203		
180min feeding	0.064	0.083	0.164	0.924	0.190	**0.027**	0.575	0.181	**0.056**	

Table S8. P-values of the paired Student's t test performed between all different zones at all different time points for the Photochemical Quenching, q_P.

qP	Before	15 m rest	15 m surrounding	15 m feeding spot	90 m rest	90 m surrounding	90 m feeding spot	180 m rest	180 m surrounding	180 m feeding spot
15min rest	**5.68E-08**									
15min surrounding	**0.033**	0.131								
15min feeding	**0.022**	**0.005**	**0.024**							
90min rest	0.628	0.409	0.212	**0.003**						
90min surrounding	0.055	0.197	0.647	**0.004**	0.099					
90min feeding	0.070	0.095	**0.024**	0.545	**0.003**	**0.030**				
180min rest	0.146	**0.019**	0.927	**0.003**	**2.93E-09**	0.700	**0.013**			
180min surrounding	0.226	0.770	0.185	**0.005**	**0.012**	**0.009**	**0.028**	0.090		
180min feeding	0.088	0.067	**0.010**	0.176	**0.002**	**0.020**	0.246	**0.011**	**0.024**	
